# Retraction: ‘Parasite prevalence and the worldwide distribution of cognitive ability’ (2010), by Eppig, Fincher and Thornhill

**DOI:** 10.1098/rspb.2024.0463

**Published:** 2024-03-06

**Authors:** Proceedings B editorial team

*Proc. R. Soc. B*. **277**, 3801–3808. (Published online 30 June 2010). (http://doi.org/10.1098/rspb.2010.0973)

Following the publication of this article, *Proceedings B* was recently made aware of potential problems with the underpinning datasets used in the analyses, which were drawn from published sources [1,2]. The editors' attention was drawn to the fact that the datasets on between-country variation in IQ had been the subject of several critiques claiming that they contain substantial inaccuracies and biases that throw substantial doubt on inferences made from them, and that these problems had not been resolved in revised versions of the dataset used by Eppig and colleagues. Upon detailed scrutiny, the editors found these claims to be convincing and asked Eppig and colleagues for their response. While the authors acknowledged at least some of the claimed flaws, they maintained that the inferences from the data were nevertheless reliable.

*Proceedings B* publishes research of outstanding scientific excellence and importance, conforming to recognized standards of scientific procedure in terms of methodology and ethical standards. Journal policy stipulates retraction where editors have clear evidence that the findings are unreliable (and may invalidate the conclusions of the paper). After carefully considering the dataset, the critiques, the authors’ response and the potential harms created by using a dataset that appears to portray human populations in some geographical regions as of below normal intelligence on average, the editors concluded that the manifest problems in the data warranted retraction in order to uphold these standards.
